# Developmental Understanding of Death and Grief Among Children During COVID-19 Pandemic: Application of Bronfenbrenner's Bioecological Model

**DOI:** 10.3389/fpsyt.2021.654584

**Published:** 2021-09-29

**Authors:** Aisha Sanober Chachar, Sana Younus, Wamiq Ali

**Affiliations:** ^1^Synapse, Pakistan Neuroscience Institute, Karachi, Pakistan; ^2^Menninger Department of Psychiatry and Behavioural Sciences, Baylor College of Medicine, Houston, TX, United States

**Keywords:** death anxiety, developmental perspective, Bronfenbrenner's ecological model, COVID-19 pandemic, child and adolescent mental health

## Abstract

COVID-19 Pandemic has influenced death-related attitudes and understanding during the childhood development leading to a life-long impact. Factors like pandemic-related movement restrictions, school closures, and parents' stay-at-home have exposed children to the phenomenon of grief and death. In that case, children anticipate adverse outcomes and fear while they struggle with unanswered questions. Children may not have coping skills needed to manage their grief in constructive ways to identify, normalize, and express their responses to the loss in their lives. Naming and validating these responses as distinctive aspects of grief process and providing safe space to express their feelings are essential components of a child's coping with loss and grief. This is crucial to consider, as different children react to and are influenced by their environments differently. This article aims to explore the developmental understanding of the process of death and grief by applying the conceptual framework of Bronfenbrenner's theory. Understanding mutual interaction between a child and various ecological systems determines how children perceive death and process grief can facilitate effective communication that has significant implications.

## Introduction

During the initial 14 months of the pandemic, more than 1.5 million children lost at least one primary caregiver (parent or grandparent) due to COVID-19-associated deaths (1). As the pandemic resulted in a substantial increase in deaths caused either directly by virus-related complications or indirectly due to limited access to health care services for chronic diseases and medical emergencies, the children's exposure to the phenomenon of death also increased exponentially (2). Children and adults differ in reacting to pandemics (3). Thus, children living through the pandemic can imbibe worries from various sources within their environment. Furthermore, these anxieties can influence each child differently depending on their developmental level of cognition and baseline understanding of death. The trauma of losing a parent or a caregiver can be a devastating life event for a child. These experiences are associated with increases in mental health conditions, substance use disorders, and chronic health conditions. To prevent further adverse consequences, the need for providing and ensuring access to evidence-based psychosocial support to these grieving children has become essential. It should not be neglected while responding to the pandemic. Thus, in assessing the grief process and children's understanding of death, it is essential to consider child's experiences, their interaction with adults, the broader culture, and exposure to losses. In addition, the child's developmental age, educational status, pre-existing mental health condition, economically underprivileged background, quarantined, and pandemics-related routine changes influence this process (4, 5). The present review uses Bronfenbrenner's bio-ecological model to discuss the contextual factors that shape the cognitive understanding and the psychological impact of death and grief process among children in the COVID-19 pandemic (Table 1).

**Table 1:** Factors affecting the process of death and grief among children in the COVID in each system of Bronfenbrenner's bio-ecological model.

**Microsystem**	**Mesosystem**	**Macrosystem**	**Exosystem**	**Chronosystem**	**Individual characteristics**
• Home environment • Parental anxiety • Parental emotional grief reaction • Parental education	• Peer conversation • Teacher's communication • Patterns of communication and support • Family values • Parenting praticesProtecting the child	• Unresponsible journalism • Cultural beliefs • Conspiracy theories • Funeral Practices	• Isolation, contact restrictions, economic shutdown, limited out-of-home leisure time activities • Limited access to healthcare income loss	• COVID pandemic • Travel ban • Changing policies	• Age, gender, temperament, and resilience • Developmental stage, i.e., cognitive, physical, psychological, social, spiritual • Death experience, trauma

## Factors Affecting Child's Understanding of Death and Grief Process

### Developmental Understanding of Death

The innate cognitive ability of a child to understand the concept of death expands during the early stages of development. This does not mean that children do not understand the notion of death. Even though their understanding is limited, they still strive to make sense of loss and grief (6). Children's ability to comprehend death and grief depends on their developmental stage, life experiences, individual temperament, parental communication patterns, and support from their environment (7). Earlier perceptions about death evolve progressively with age. Speece and Brent (8) identified these concepts according to children's cognitive representation of death (Table 2). According to these theoretical concepts, for a 4-year-old, death is reversible, which means if someone has died, they can come back to life. Children also do not fully conceptualize the fact that they are mortal. Instead, they believe death happens to older people and, in some instances, evil people. By the age of 8–10, children now comprehend personified death as an unavoidable life event.

**Table 2:** Developmental stages during childhood and cognitive representation of death.

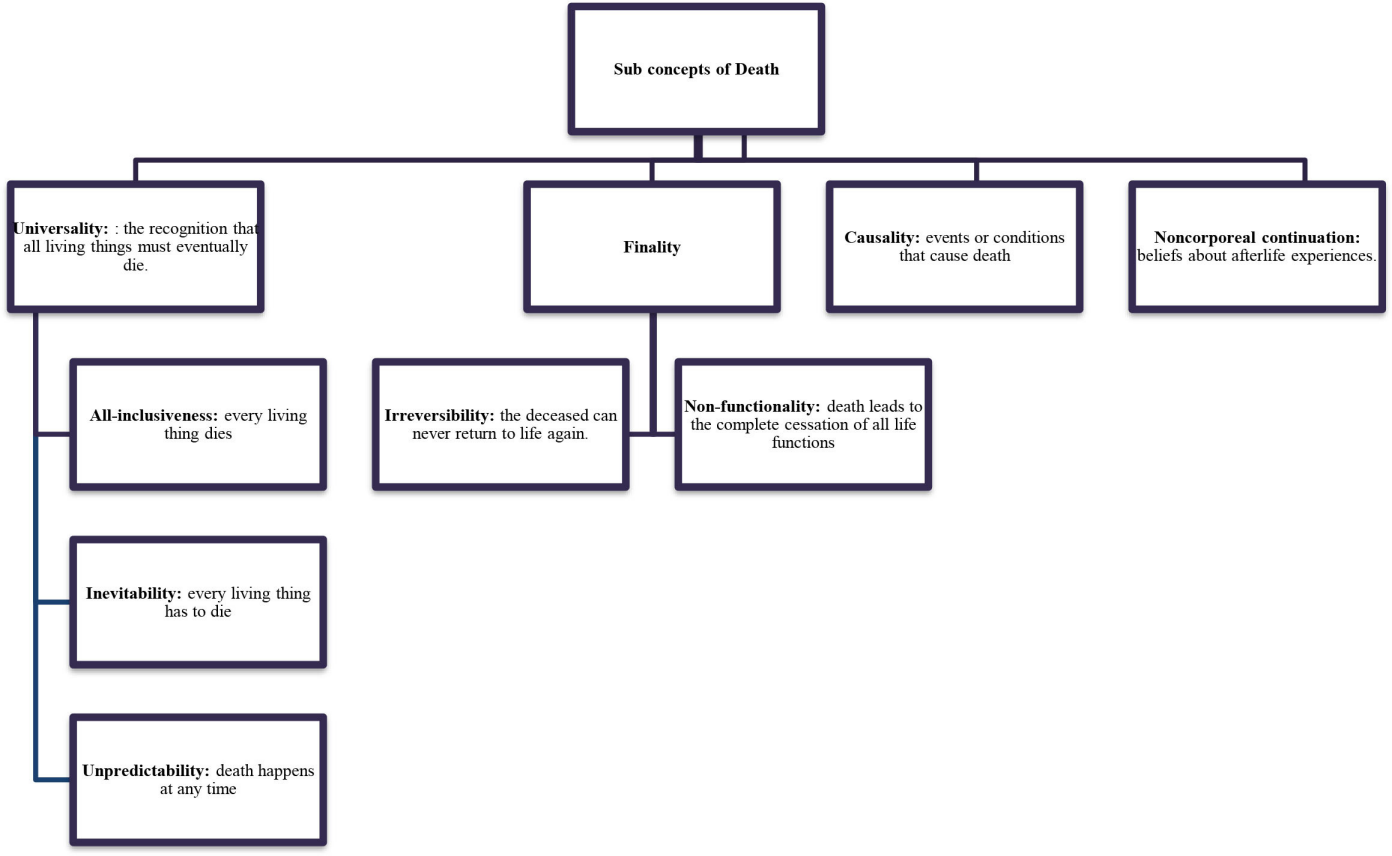

### Death-Related Emotional Responses

Emotional responses to death include uncertainty, fear, and anxiety. These responses depend upon an individual's comprehension and appraisal for threat, meta-cognitive beliefs, intolerance of uncertainty, cognitive biases toward physical symptoms, and existential concerns, including death and dying (9). Uncertainty is an inability to determine and predict the meaning of ongoing events. It can be a result of ambiguity, i.e., conflicting, incomplete, or inadequate information; complexity, i.e., difficult to understand information; and unpredictability, i.e., likelihood or risk of the future outcome of the event (10). When faced with uncertainties, children and families might feel paralyzed and struggle with making urgent or everyday decisions (11). Fear is a powerful emotion that plays an essential role in survival and induces a fight or flight response. Regardless of one's response to fear, it thrives on unfamiliarity and lack of information. It can be spontaneous or deliberate (12). It can also invoke a response to anticipated threats or thoughts about potential dangers, generally manifested as anticipatory anxiety. These emotions in current times can arise due to existential threats, uncertainty about a disease process, available treatments and vaccinations, and loss of meaning and coherence. In some instances, anticipatory fear may precede an actual pandemic. Subsequently, these reasons impact one's behaviors, cognitions, emotions, and interpersonal skills.

### Fear of Death, Death Anxiety, and Death-Related Attitudes

Fear of death is a conscious phenomenon specific to dying. Reasons to fear death include loss of self, fear of the unfamiliarity, pain, suffering, and leaving family members in distress (13). In contrast, death anxiety is considered to be unconscious and more generalized in nature. Death anxiety includes avoidance and acceptance. Death avoidance is an attitude toward death is when a person attempts to avoid thinking or talking about death. Note that death avoidance differs from fear of death which results from confrontation with death and the emotions attached to it (13). Whereas, death acceptance is the ending and desirable stage of coping with death itself (14). It is a psychological readiness for the final separation from life that consists of the cognitive awareness of mortality and a positive emotional reaction to that awareness (13, 15). Different attitudes represent death acceptance. The first is neutral acceptance, in which a person perceives and accepts death as an integral part of life. They are not afraid of death but, at the same time, do not welcome it. The second attitude is approach acceptance, in which a person perceives death as a passage to a better afterlife. This attitude is significantly associated with religiosity (16). The third attitude is escaping acceptance, where a person perceives death as an escape from the pain of life, thus, a desirable alternative to life.

The question arises if the fear of death exists in most children who have not developed a fully mature understanding of death. In some instances, fear of death increases in early adolescence (17). This perspective suggests that even when a child achieves a mature understanding of death, additional factors influence fear of death. Developing a mature concept of death could affect children's fear of death in two ways. Either it could provoke it, or it could reduce it; the latter leads to reduction in child's struggle with unanswered questions about their earlier conceptualization of death. It remains unclear if interventions targeted to reduce fear of death would benefit those children who have mastered all five subcomponents of the death concept as some fear remains, even after achieving a mature understanding of death (18). Further research to investigate which possible developmental scenario would prove beneficial has significant implications for clinical and educational practices.

### Terror Management Theory

Terror management theory (TMT) proposes that death anxiety drives human behavior (19). TMT reasons that death reminders increase the potential for experiencing existential anxiety, and can increase mental health symptoms (20). Continuing the TMT principle, Morality Salience (MS) theory highlights the importance of innate belief systems to protect oneself from existential anxiety. Situations that demand confrontation to one's mortality, as evident in the current pandemic; there is an increased need to hold on to the existing belief system. Hence, any counter-narrative appears as a threat and any reinforcement of the current belief system provides psychological safety. During a threat to survival and prosperity, humans shift toward the collectivistic mindset (21). These theories suggest that the fear of death drives people to maintain their cultural beliefs at the expense of health related safety. Collective beliefs shared by the community can affect individuals' behavior or emotions when experiencing stress. This is termed “***milling***,” described by *Contagion theory* (22). During *milling*, people become incredibly conscious of the crowd's attitudes and respond by adopting them to avoid external ridicule. Independent actions reduce through *milling*, and new behavioral patterns emerge from extreme collective behavior observed during the pandemic. These factors, directly and indirectly, aggravate the anxiety associated with the uncertain pandemic times for children and families.

## Bronfenbrenner's Model of Child Development During COVID-19

**Figure 1:**
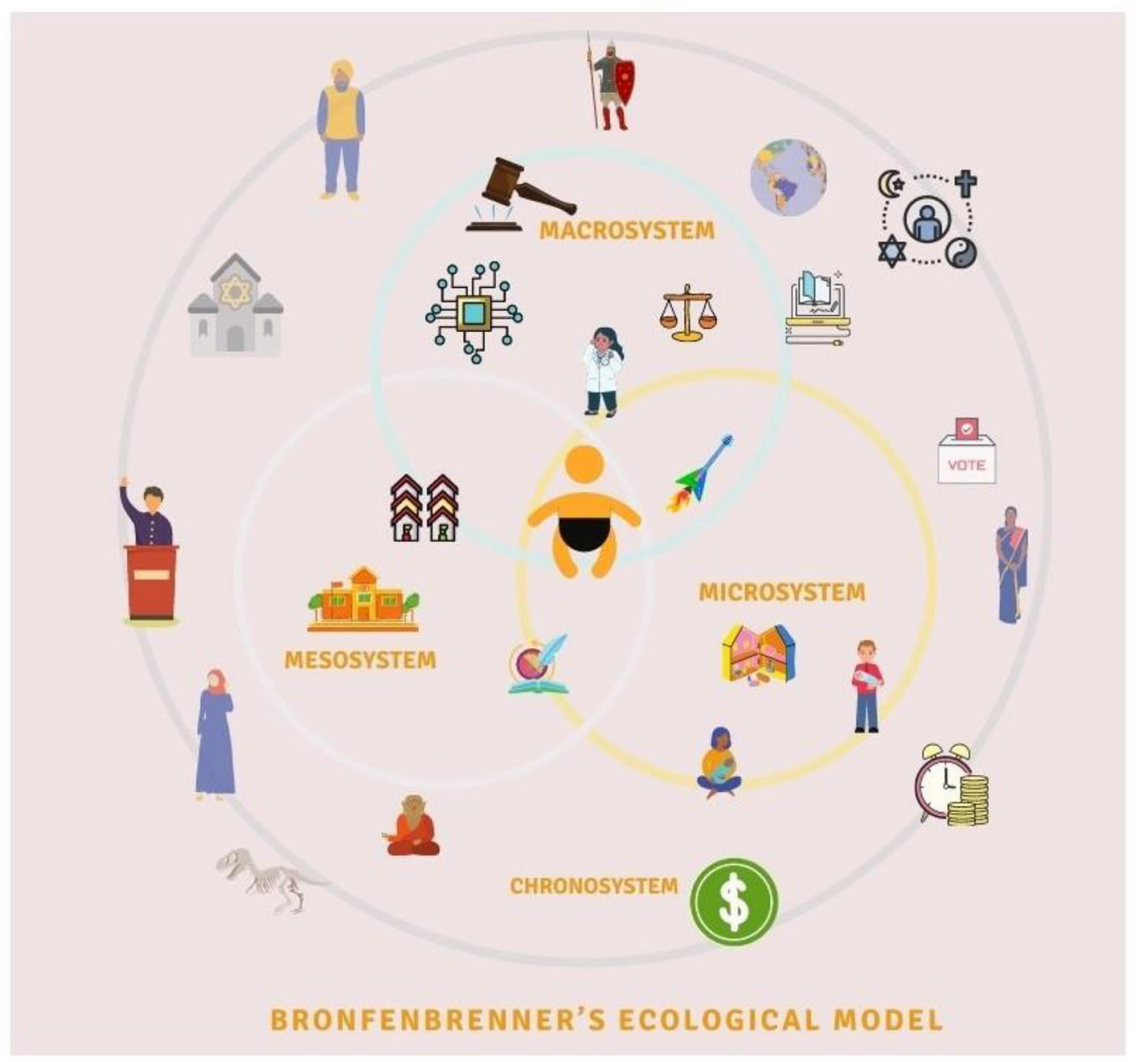
Bronfenbrenner's bio-ecological systems and their interactions with childhood development.

**Figure 2:**
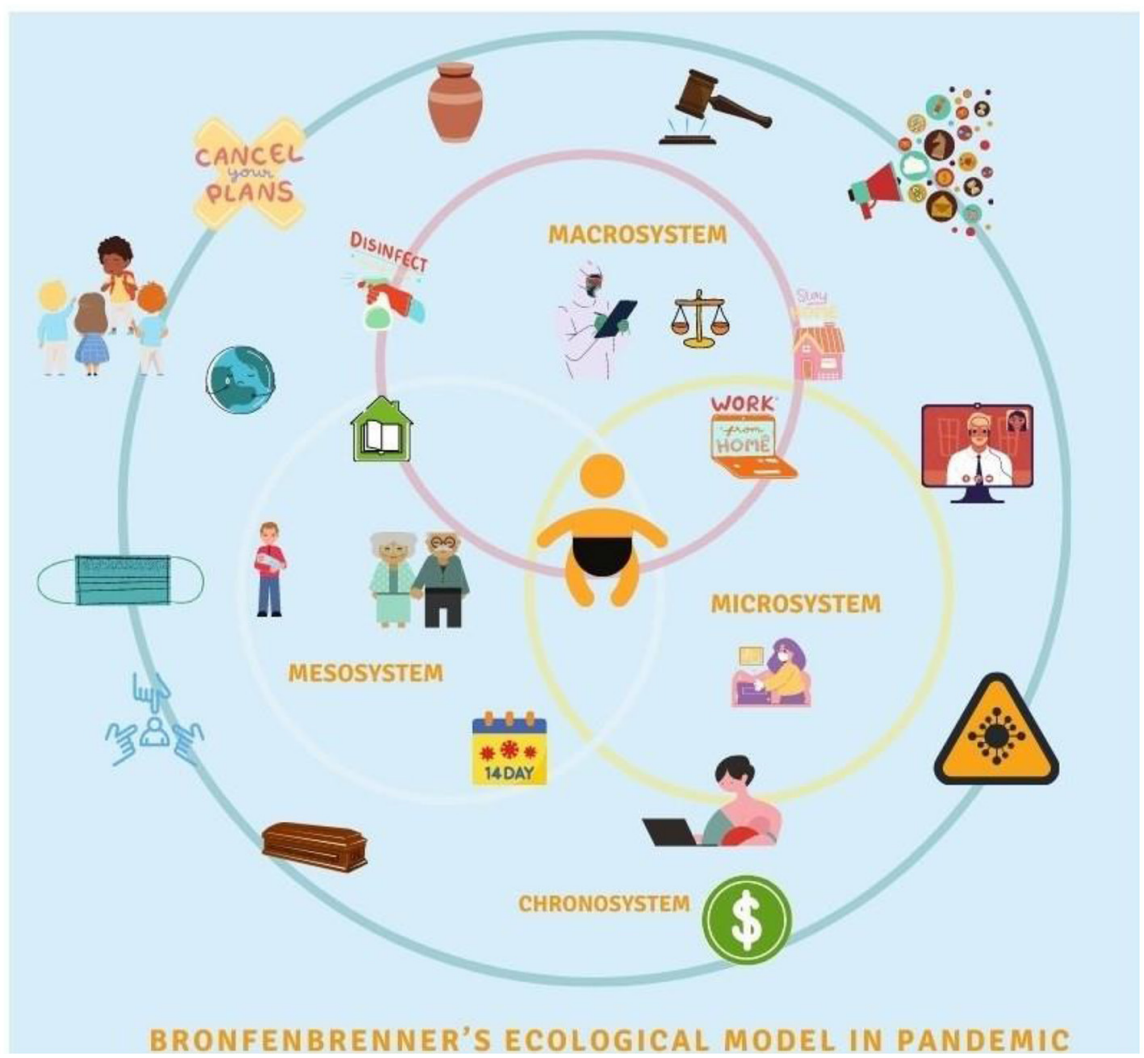
Bronfenbrenner's bio-ecological model; systems and their interactions with childhood development during COVID-19 pandemic.

Urie Bronfenbrenner's bio-ecological model provides a framework to describe how a child's innate qualities and environments influence growth and development in different settings (23). This model maintains the child at the central core with five encompassing systems, ranging from the most intimate to the broadest (Figure 1). These systems and their interactions with childhood development are crucial regardless of pandemic times. Nonetheless, the COVID-19 pandemic has further complicated the interactions between these systems at all levels (Figure 2). Thus, it is valuable to apply Bronfenbrenner's bio-ecological model to understand pandemic's impact on grief and death process among children.

The ***microsystem*** is children's immediate environment, including school, day-care, home, friends, and the local community or immediate neighborhood. The interactions between these constructs in the microsystem and the child's reaction toward them directly affect childhood development. The large magnitude of the COVID-19 pandemic has affected children in many ways. Although children appear to have lower mortality rates directly from COVID-19, the indirect impact has resulted in a rise in child mortality rates mainly due to the limited access to health services, immunization, and antenatal care; reversing decades of progress toward eliminating preventable child deaths globally (2, 24). On the other hand, the number of children losing a primary caregiver due to COVID-19 associated deaths is overwhelming (1). Subsequently, as the pandemic evolves, attempts are made to track the number of grieving children for each COVID-19 associated death (25). Sudden parental death secondary to COVID-19 can profoundly affect grieving children, putting them at elevated risk of traumatic grief, mood and anxiety disorders, poor educational outcomes, unintentional death or suicide, and consequences that can persist into adulthood (26).

Children who have not directly experienced the death of their loved ones have also been affected by the pandemic. Whether cohesive or disintegrated, the existing family system is compromised due to changes in routine and the introduction of online teaching. The risk of maltreatment, violence at home, and poor nutrition also increase for many vulnerable children. Children from disadvantaged backgrounds are more prone to being affected by the consequences of the COVID-19 outbreak. They are predisposed to poor health outcomes, lower physical activity levels, suboptimal diet, greater disengagement from school, lower academic performance, and more socio-emotional difficulties (27). Families' reactions to the pandemic-related anxieties range from fear to indifference to fatalism (3, 28). Some families may disregard the risks and fail to engage in recommended health behaviors such as vaccination, hygiene practices, and social distancing. Others may react with intense anxiety or fear. Parents with anxious temperaments are likely to fear contagion and view the world as an existential threat, described in the literature as health anxiety and experience anxiety, respectively (29, 30). However, a moderate level of fear or anxiety encourages people to cope with health threats, but severe distress can be debilitating. This diversity of coping styles among families stems from their unique challenges. During the pandemic, the lack of support for parents from extended family members and other social support systems results in high levels of parental emotional distress (31).

The ***mesosystem*** includes the interaction of different microsystems. For example, it involves the connections between school and home, family and community, and between friends and family. During the pandemic, as children interact within various elements of their microsystem, information exchange during these interactions leads to an alteration of perception and attitude related to death. For instance, household conversations about vulnerability and risk associated with exposure for older family members, limited visitations to grandparents, health communications in schools, and outdoor activities can raise curiosity in children. This curiosity, if addressed, can lead to a better understanding of the situation. However, not addressing it can lead to confusion and invoke fear due to uncertainty. This multidimensional exchange multiplies the extent and impact of unanswered queries. There is uncertainty in the early stages of a pandemic, especially when outbreaks come in waves, with schools and workplaces closing for the while and reopening later in-person or in a hybrid fashion (32). As the curve flattens, a single incident of viral exposure disrupts the entire mesosystem. The cost of each exposure is multilayered. For a child exposed in school, one significant consequence is the cessation of in-person learning that is also emotional in nature. Blaming oneself as a source of illness, anger toward other children for not taking optimal precautions, and guilt associated with being responsible for the morbidity and mortality of someone else are some of the emotional reactions exhibited by children. On the other hand, parents worry about the risk of illness and associated fear of death compounded by stress associated with mobilization of resources for childcare as they manage in-person and online, work, and schooling (31).

The ***exosystem*** refers to the relationship between two or more settings that influences children's development. It includes available health services, employment opportunities, or neighborhood safety. Disruption in the exosystem during the pandemic adversely affects children as lockdown measures reduce opportunities for children to participate in extracurricular activities, which serves as a coping outlet for many children. Contact with supportive school staff, community members, and access to the justice system and child protection services are also decreased due to policies related to in-person gatherings (28). Children are also indirectly affected by elements of this system as their families are impacted by the economic shutdown leading to unmet basic needs (33).

The healthcare system is one of the most critical elements in this system that significantly impacts children's death-related attitudes. For an overall healthy child, pandemic disrupts annual physical exams, dental visits, eye exams, and routine vaccinations. In addition, emergent or urgent visits to the emergency rooms or clinics are associated with significant anxiety related to fear of exposure to virus during the visit. For children with comorbidities, routine hospital visits are associated with similar anxieties. However, these are magnified by new health care policies during visits, limitations associated with the number of adults accompanying children in health care settings, and contact precautions leading to limited access to resources like screens and toys. For children who require home healthcare services, nursing care is complicated by contact precautions and physical distancing. Despite common fear of shots, doctors' visits, and dental exams, health care settings are generally considered places where one gets help. During a pandemic, the fear of contamination associated with health care providers and health care settings can distort this image. This can be disorienting for children far from the developmental phase of identifying abstract and conflicting ideas.

Media is another crucial component of the exosystem. In the past, movies and TV shows have toned down death by immunizing the masses against its emotional relevance by exposing them to the frightening nature of pandemics in the context of entertainment (34). Nevertheless, to view a past pandemic in a movie is different from viewing live reporting of death tolls from one's lifetime; the latter is broadcasting one's reality which can have adverse outcomes. Moreover, daily exposures to death stats can cause emotional numbness in some, whereas it can trigger anxiety for many. This concern led to a media release by WHO declaring an “infodemic” crisis (35). Media has the potential to impact the development of children in positive ways. As the anxiety associated with health care settings rise in children and families, they also observe the collective effort of first responders and health care providers to provide care for those in need at the cost of their personal safety. On the one hand, as media portrays the deficiencies and incompetence of governmental organizations and highlights the daily surge in cases and deaths, it also showcases the strength of communities in these difficult times. These and many other examples of unity serve as tools used by caregivers to alleviate illness and death-related anxiety.

***Macrosystem*** are “blueprints” for interlocking social forces and their interrelationships in shaping child development. It consists of culture, beliefs, ideas, and the political and economic system. They provide broad ideological and organizational patterns that are not static but might change throughout time due to economic, geopolitical, and technological changes. Children's perception of death-related attitudes are also formed by their cultural values and geographical location. For instance, death experiences for children in a war zone area is related to witnessing daily violence. Their death attitudes differs from those living in peace and prosperity (36). This has a relevance as majority of the children affected by COVID-19 associated deaths are from countries with ongoing political unrest, thus, complicating the grieving process (25).

The political and economic system plays a significant role during the pandemic. As the outbreak spreads globally, each country becomes a key player in its containment and management. As the global political struggles continue to control the spread of the virus, inequities in the allocation of health care funding worsen the economic burden in various countries. Closure of businesses, travel ban, limited financial support and allowances by governments, layoffs from jobs further added to this burden.

COVID-19 pandemic has high morbidity and mortality rate. As the death toll mounts, the compounded weight of pain and loss disrupts the ability to grieve, affecting cultures and faiths worldwide. Communities mourn alone with nationwide lockdowns while taking care of their sick (3). Each culture and religion has a different explanation and response to the pandemic that occurs with collective denial of impermanence and mortality in the background. The attitude toward precautions and treatment varies according to these cultural explanations. In many cultures, people seek help from ancestral spirits and trust their elders and traditional healers, seeing them as the custodians of culture who guide them regarding life's conduct (37). People tend to resort to their culturally prescribed religious practices to cope with the anxiety stemming from the existential threat of the pandemic. Social and physical distancing makes these difficult as most religious practices comprise congregational rituals and ceremonies. The inability to meet with trusted fellow community members and praying together to preserve the sense of cohesiveness during times of uncertainty is distressful especially on religious occasions. Easter, Eid, Thanksgiving, Halloween, Holi, Diwali, Hajj, Christmas, Passover, and Hanukkah are observed unconventionally while adults resort to innovative ways to maintain a sense of cohesiveness. Holidays are vital to create valuable experiences for children, as they benefit from these times by developing social skills, interacting with others, and sharing their values with peers belonging to other faiths and cultures. Unfortunately, the pandemic compromises these practices and their outcomes in different ways.

Pandemic-related conspiracy theories complicated the situation further. For instance, the “Chinese virus” narrative originated from the United States and quickly spread to the entire world. The geopolitical landscape perpetuated racial discrimination against Asians ranging from structural and political to professional and personal levels. This contributed to emotional challenges for children of Asian descent and their peers living across the world. These perceptions arise during uncertainty, especially if the circumstances are threatening and personally relevant (38). Belief in conspiracy theories happens to be a culturally universal phenomenon (39). Believing that it is a “man-made virus” with bioterrorist intent, and vaccination induced infertility were some of the conspiracy theories that were the highlights of the year 2020.

***Chronosystem*** includes the context and dimension of time, including both change and constancy in the children's physical and social environment such as family, neighborhood, country, culture, and historical time. It also includes intangible factors such as values, customs, and ideals.

Among many religions, a widely practiced ritual is washing the dead body with bare hands and spending time with the dead body. Burial practices like these are among the most significant modes of spreading the disease and hence highly discouraged during the pandemic (40). The community's inability to bury the dead according to cultural and religious practices due to shortages of coffins and insufficient funeral staff can result in unresolved grief with hindered death-related closure (41, 42). Managing a loved one's funeral is an emotional time for bereaving families, and abandonment of traditionally prescribed practices can be perceived as dehumanizing at this challenging time (43, 44). As families struggle through the process of grieving and closure, children are impacted by their prolonged and unresolved grief which can manifest as confusion about the death of the family members, questions about the cause and meaning of death, and anxiety associated with their death and the possible death of living caregivers (45–47).

## Implications

The exposure to death of a loved one or a threat (perceived or actual) toward their safety can interfere with the child's cognitive understanding of death. Below are the critical implications for various stakeholders keeping the view of Bronfenbrenner's ecological model.

### Children's View of Pandemic

Children fear parental safety more than personal safety in the current context. They face uncertainties about being infected, the seriousness of the infection, the optimal type of treatment or protective measures, and whether a pandemic is truly over. Children may also express concern about possible infection, a threat to family integrity, separation from their school friends, and, more importantly, about death. As a result, they manifest anger, restlessness, frustration, and disinterest (48). While adults are busy mitigating the risks and combating the crisis, the message to children translates as, “you are safe as long as you are indoors.” Reassurance of this kind does not help the child's fear of parental safety. Instead, this account can also induce feelings of guilt related to being safe in the midst of a crisis. Many adults firmly believe that children are too young to suffer, understand, internalize, and remember traumatic events to which they are exposed. Therefore, they try to protect children from the emotional discomfort that death induces. There is a “general multi-determined tendency in too many adults to encourage children to deny and repress painful effects and threat-perceived real events, often to the child's detriment” (49). The need to mourn effectively and sufficiently is as much a right of children as that of adults. However, this need must match the child's developmental ability and tendency to regulate emotions with parental emotional availability (50). Resilience allows adaptation in the face of adversity and trauma while enduring the pain and distress (51). Practicing strategies like cultivating a positive self-image, maintaining hope, and keeping things in perspective can nurture resilience (52). Children respond well to reassurance and accurate and timely information about the environment's isolation status and expected changes. Empowering children in quarantine and isolation by including them in the decision-making process helps restore dignity and a sense of self-worth in challenging situations (53).

### Child's Grief Reactions

Acute grief reactions vary with symptoms unique to the loss experienced, which can be painful and impairing but do not necessarily represent a mental illness (54). Unfortunately, these reactions are not fully understood due to a lack of recognizing secondary stressors integral to the bereavement experience (55). The child not only deals with the loss of the deceased person but also worries about adjustments concerning various aspects of life; adaptation to loss involves restoring coherence to the narrative of the child's life (56).

For adults, grief is typically an unusual and disturbing experience. It is more so for children who may have even less understanding of what is happening or can happen to them. The child's cognitive and emotional development influences these reactions. For some children, grief comes in relatively short bursts over an extended time. This uneven or intermittent course of suffering is one of the most exclusive and typical childhood bereavement features. Some children may experience more intense grief responses. Preschool children, may exhibit clinging and dependent behaviors, phobic reactions, sleep and appetite disturbances, nightmares, loss of bladder and bowel control, temper tantrums, and hyperactivity (57). The younger school-age children may not express psychological suffering but manifest it through play and behavioral symptoms. They often have sleep, appetite, and concentration disturbances, somatic complaints, irritability, hyperactivity, decline in school performance, and sibling rivalry. Older children can express unpleasant internal emotional states verbally. These include anxiety, panic, dysphoria, maladaptive behaviors, such as aggression and interpersonal conflicts. The adolescents may present with depressed mood, social withdrawal, suicidal ideation and behavior, defiance, impulsive behavior, and substance use (57).

### Communication

Bereaved children and family members may also experience intense survivor's guilt. Support from a trusted spiritual leader, a therapist, or grief support groups play a therapeutic role and enhance a sense of social connectedness (58). Storybooks are a helpful tool to acquaint children with communication about death and grief (59). Feraco et al. (60) proposed four main approaches for caregivers: Acknowledge, Discuss, Do, and Reflect. A discussion of this kind with the child often helps them express some of their concerns and promotes trust, understanding, and control over the situation. Key aspects to consider while communicating with children are as follows:

- Clarify the child's knowledge and identify the source of concern while discussing the pandemic.
- Provide repeated opportunities for the child to talk about their worries, questions, and feelings about the pandemic and how it affects them. One may not always need to have an answer, but active listening can go a long way.
- Keep the process as a dialogue rather than a statement.
- Keep the conversation hopeful even when the child knows understands the gravity of the situation.
- Ensure them that the health care providers continue to take measures to combat this situation.
- Communicate the facts and ensure an adult's presence to keep them safe.
- Limit media exposure to prevent overwhelmingly inaccurate information.

### School

School refusal may occur at any time but is most common in children ages 5–7 and 11–14 while transitioning to elementary and middle school, respectively. In non-crisis times, school refusal often begins following a period at home when the child becomes closer to the parent, such as a holiday break, long weekend, brief illness, the death of a pet or relative, or change in schools. These children suffer from a paralyzing fear of leaving the safety of their parents and home. Subsequently, prolonged online schooling in the face of pandemic can potentiate school refusal. Therefore, there is also a need for flexible and preventative school-based interventions to enhance children's understanding of loss and address misconceptions about the death and grief process (61). Schools and teachers must aim to provide information about death, offer an appropriate vocabulary; present diverse cultural perspectives; allow the elaboration of eventual losses and offer help to manage grief, demystify media contents and myths; and reflect upon the elements of funeral rites to integrate spiritual and religious perspective (47).

### Health Care Professionals

Health care professionals working with bereaved children should consider children's views of death in their communications during clinical encounters. They can help children to rework their understandings of death as they progress developmentally. The main tasks for grieving children include understanding the loss associated with the state, expressing emotions and strong reactions, remembering the deceased, and learning the integration of the loss in one's life (62). This approach provides opportunities for growth and the realization of “good grief” in the context of irreversible loss and unavoidable sadness. In addition, clinicians should consider exploring children's understanding and needs as part of the management process. These could include: gathering adequate information about fears and anxieties, providing reassurance to reduce child's guilt and self-blame, empathetic listening and validation of child's feelings, helping them with overwhelming emotions, involving them in decision making, promoting routine activities, assisting parents in modeling their grief behaviors and giving the child opportunities to remember the deceased one or talk about them. Additionally, the clinical approach must incorporate cultural and contextual factors that may limit the spread of contagion (63). Therefore, it is crucial is to incorporate traditional religious practices and cultural beliefs that can significantly contribute to clinical challenges while managing the pandemic (64).

## Conclusion

COVID-19 Pandemic would influence death-related fears on childhood development, impacting them for years of their life. A bioecological perspective can help us understand the differences between how children's understanding of death is impacted or influenced by their environments compared to adults. This is crucial to consider, as different children react to and are influenced by their environments differently. Adults must refrain from having a predetermined opinion of how a child must respond to a situation over a given period. Bereaved children and adolescents may not have coping skills needed to manage their grief in constructive ways. Therefore, like all bereaved adults, children need to identify, normalize, and express their responses to the loss in their lives. Naming and validating these responses as distinctive aspects of grief process and providing safe space to express their feelings are essential components of a child's coping with loss and grief. These experiences become overwhelming when children are led to view them that way. Nothing is scarier for a child than their feeling of helplessness and fear of losing a loved one.

## Data Availability Statement

The original contributions presented in the study are included in the article/supplementary material, further inquiries can be directed to the corresponding author/s.

## Author Contributions

AC and SY: review of literature for the work, critical revision of the work for important intellectual content, design of the work, drafting of the manuscript, final approval of the version to be published, and agreement to be accountable for all aspects of the work. WA: drafting the manuscript, final approval of the version to be published, and agreement to be accountable for all aspects of the work. All authors contributed to the article and approved the submitted version.

## Conflict of Interest

The authors declare that the research was conducted in the absence of any commercial or financial relationships that could be construed as a potential conflict of interest.

## Publisher's Note

All claims expressed in this article are solely those of the authors and do not necessarily represent those of their affiliated organizations, or those of the publisher, the editors and the reviewers. Any product that may be evaluated in this article, or claim that may be made by its manufacturer, is not guaranteed or endorsed by the publisher.
